# Potassium Channels in the Uterine Vasculature: Role in Healthy and Complicated Pregnancies

**DOI:** 10.3390/ijms23169446

**Published:** 2022-08-21

**Authors:** Wyanet Bresnitz, Ramón A. Lorca

**Affiliations:** Division of Reproductive Sciences, Department of Obstetrics and Gynecology, University of Colorado Anschutz Medical Campus, Aurora, CO 80045, USA

**Keywords:** pregnancy, uterine artery, uteroplacental circulation, endothelial cells, smooth muscle cells, vasodilation, uterine artery remodeling, potassium channels, BK_Ca_ channel, K_ATP_ channel, Kv channel, intrauterine growth restriction, pre-eclampsia, pregnancy-induced hypertension, gestational diabetes mellitus

## Abstract

A progressive increase in maternal uterine and placental blood flow must occur during pregnancy to sustain the development of the fetus. Changes in maternal vasculature enable an increased uterine blood flow, placental nutrient and oxygen exchange, and subsequent fetal development. K^+^ channels are important modulators of vascular function, promoting vasodilation, inducing cell proliferation, and regulating cell signaling. Different types of K^+^ channels, such as Ca^2+^-activated, ATP-sensitive, and voltage-gated, have been implicated in the adaptation of maternal vasculature during pregnancy. Conversely, K^+^ channel dysfunction has been associated with vascular-related complications of pregnancy, including intrauterine growth restriction and pre-eclampsia. In this article, we provide an updated and comprehensive literature review that highlights the relevance of K^+^ channels as regulators of uterine vascular reactivity and their potential as therapeutic targets.

## 1. Introduction

### 1.1. Maternal Vascular Changes during Healthy Pregnancies and Pregnancy Complications

Pregnancy initiates major changes in the maternal uterine circulation that allow for the transfer of nutrients and oxygen to the developing fetus. During pregnancy, the uterine vasculature undergoes a process that includes marked vasodilation, increases in the lumen and length of the main uterine artery (UtA), as well as the downstream arcuate and myometrial arteries, and the remodeling of the spiral arteries along with the distal portion of the myometrial arteries [1]. The increased vasodilation and vascular remodeling serve to decrease the vascular tone and raise UtA blood flow, resulting in directing the major portion of the pregnancy rise in cardiac output to the uteroplacental circulation, which equates to approximately 1 L/min or 20% of cardiac output near term [2].

Many factors contribute to the increased vasodilation of uterine vasculature during pregnancy, including those affecting endothelial and vascular smooth muscle cell function. For instance, endothelial nitric oxide (NO) is a key mediator for the vasodilation prompted by shear stress, estrogen, or increased vascular endothelial growth factor production, and also for modulating circumferential remodeling [3,4,5,6,7]. Estrogen is a sex steroid hormone that is known to increase both locally and systemically during pregnancy and to cause the vasodilation of uterine vasculature and thus increase UtA blood flow [8] via acting to increase endothelial NO synthase expression and activity [9,10]. Estrogen also has potent effects in the UtA smooth muscle, where it can activate K^+^ channels to induce vasodilation [11]. Vascular remodeling during pregnancy results from tissue and cell hypertrophy, endothelial and vascular smooth muscle hyperplasia, and matrix remodeling (reviewed by [1]).

Abnormalities in these pregnancy-associated vascular dilation and remodeling processes are associated with the pregnancy complications of intrauterine growth restriction (IUGR) and pre-eclampsia, in which, the normal vasodilatory effects of acetylcholine (ACh), bradykinin, NO, endothelium-derived hyperpolarizing factor (EDHF), and thromboxane-mediated responses are impaired [12,13,14,15]. In addition, chronic hypoxia is one of the most common insults to fetal development, thought to be an underlying contributor to and raising the risk of IUGR and pre-eclampsia [16,17]. Chronic hypoxia has been shown to impair the reduction in vascular tone, uterine vasodilation, and growth occurring during normal pregnancy, which results in a lower rise in UtA blood flow compared to pregnancies occurring in a normoxic environment [17,18,19,20]. Furthermore, although not within the scope of this review, the placenta also plays important roles in the pathogenesis of vascular complications of pregnancy, as has been reviewed previously [21].

### 1.2. K^+^ Channels as Regulators of Vascular Function

K^+^ channels are membrane proteins that allow K^+^ ions to move across cell membranes following an electrochemical gradient, thus hyperpolarizing these membranes and, in turn, regulating a wide variety of cellular processes, including the control of cell excitability, contraction, and proliferation [22,23]. Particularly important are the K^+^ channels’ contribution to vasodilatory responses in resistance arteries. Specifically, the membrane hyperpolarization induced by K^+^ channel activation in smooth muscle cells leads to closing voltage-gated Ca^2+^ channels and a reduction in Ca^2+^ entry, thereby preventing contraction [24]. In endothelial cells, K^+^ channels are involved in EDHF responses, independent of NO and prostaglandin (PG) production [25]. It has been proposed that the hyperpolarization of endothelial cells further increases the entry of Ca^2+^ by increasing its electrochemical gradient [26]. Additionally, K^+^-channel-dependent endothelial cell hyperpolarization can also be propagated to the smooth muscle via gap junctions [27]. Thus, K^+^ channels tightly regulate the balance between pro-contractile and pro-dilatory responses, which is key for the proper function of vascular vessels and their response to different physiological conditions. In the uterine vasculature, the vasodilatory roles of K^+^ channels seem to be critical for the rise in UtA blood flow during pregnancy. Multiple K^+^ channel types have been described to participate in this process, including Ca^2+^-activated, ATP-sensitive, and voltage-dependent K^+^ channels.

**Figure 1:**
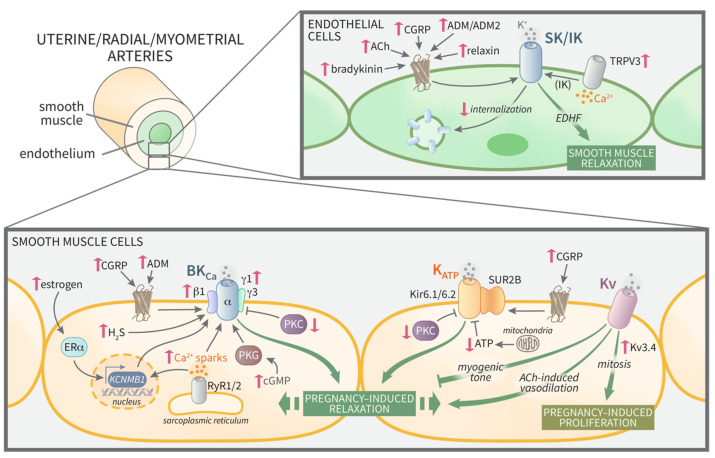
Mechanisms underlying the modulation of K^+^ channels in the uterine vasculature during normal pregnancy. Pregnancy increases uterine blood flow by augmenting vasodilation and/or remodeling of uterine, radial, and myometrial arteries. A critical mechanism for increased vasodilation is greater activation of K^+^ channels in the endothelium (**top panel**) and smooth muscle (**bottom panel**) of these uterine blood vessels. Pink arrows show the effects of pregnancy. ACh, acetylcholine; ADM, adrenomedullin; ADM2, adrenomedullin 2; ATP, adenosine triphosphate; BK_Ca_, large-conductance Ca^2+^-activated K^+^ channel; β1, BK_Ca_ β1 subunit; cGMP, cyclic guanosine monophosphate; CGRP, calcitonin gene-related peptide; EDHF, endothelial-derived hyperpolarizing factor; ERα, estrogen receptor α; γ1 and γ3, BK_Ca_ γ1 and γ3 subunits; H_2_S, hydrogen sulfide; IK, intermediate-conductance Ca^2+^-activated K^+^ channel; K_ATP_, ATP-sensitive K^+^ channel; *KCNMB1*, BK_Ca_ β1 subunit gene; Kir6.1/6.2, inward-rectifier K^+^ channel type 6.1 and 6.2; Kv, voltage-gated K^+^ channel; Kv3.4, voltage-gated K^+^ channel type 3.4; PKC, protein kinase C; PKG, protein kinase G; RyR1/2, ryanodine receptor type 1 and 2; SK, small-conductance Ca^2+^-activated K^+^ channel; SUR2B, sulfonylurea receptor type 2B; TRPV3, transient receptor potential vanilloid type 3.

In the present article, we discussed the current literature available supporting the important role of these K^+^ channels in the uterine vascular adaptation to pregnancy under healthy conditions and how the impaired function of these channels is related to different pregnancy complications. The information provided in this article may be relevant for researchers aiming at elucidating the complex factors underlying the pregnancy-dependent changes in uterine vasculature and for the identification of candidates to develop therapeutic approaches to prevent or alleviate pregnancy complications associated with impaired uteroplacental perfusion. A summary of the pregnancy-dependent regulation of K^+^ channels in the uterine vasculature described below is shown in Figure 1.

## 2. Ca^2+^-Activated K^+^ Channels

Ca^2+^-activated K^+^ channels are ubiquitously distributed throughout the body, where they integrate intracellular Ca^2+^ signals with the regulation of the membrane potential. There are three main subfamilies of these channels: large conductance (BK_Ca_, Slo1, K_Ca_1.1), intermediate conductance (IK, K_Ca_3.1), and small conductance (SK, K_Ca_2.x) Ca^2+^-activated K^+^ channels [28].

The pore-forming BK_Ca_ channel α subunit is encoded by a single gene (KCNMA1). Each α subunit consists of seven transmembrane domains with an extracellular N-terminus and a large cytosolic C-terminus [29,30]. Tetramers of α subunits form functional channels, which can be associated in a 4:4 stoichiometry with two types of auxiliary proteins (β or γ), for which, four β (β1–β4) and four γ (γ1–γ4) have been described [31]. In the vasculature, BK_Ca_ channels are predominantly expressed in smooth muscle cells, where β1 and γ1 auxiliary subunits have been shown to increase BK_Ca_ activity [32,33,34].

The IK channel is encoded by the KCNN4 gene (K_Ca_3.1, IK1, SK4), whereas the SK channels are encoded by KCNN1 (K_Ca_2.1, SK1), KCNN2 (K_Ca_2.2, SK2), and KCNN3 (K_Ca_2.3, SK3) genes. The pore-forming α subunits of IK and SK channels have six transmembrane domains with both N- and C-terminus cytoplasmic. The IK and SK channels have often been described as occurring in endothelial cells [35].

### 2.1. BK_Ca_ Channels in Healthy Pregnancies

In the uterine vasculature, as in other vascular beds, BK_Ca_ channels play important roles in promoting vasodilation and reducing myogenic tone. Specifically, pregnancy progressively raised BK_Ca_ channel activity in UtA smooth muscle cells isolated from pregnant sheep and mice compared to non-pregnant sheep and mice [36,37]. Moreover, in UtA from sheep, myogenic tone was increased by blocking BK_Ca_ channels with tetraethylammonium (TEA) in vessels from pregnant animals, but not from non-pregnant animals [36]. Similarly, the vasoconstrictor responses to the BK_Ca_ channel blocker iberiotoxin (IbTX) were augmented throughout pregnancy in mouse UtA [37]. Highlighting the important contribution to UtA blood flow, the in vivo infusion of the BK_Ca_ channel blocker TEA decreased UtA blood flow in pregnant sheep [38]. Knockout of the BK_Ca_ α subunit reduced the increase in UtA diameter during pregnancy and the number of pups per litter [37]. However, UtA resistance in vivo was not impacted [37], suggesting that other factors may compensate for the absence of BK_Ca_ channels in vivo.

The pregnancy-related changes in BK_Ca_ channel function are not likely due to an altered protein expression of the pore-forming α subunit. An increased expression of BK_Ca_ channel β1 and γ1 subunits has been described, although the α subunit expression did not change during pregnancy [36,37,39]. Furthermore, the protein expression of β1, γ1, and γ3 subunits has been described in whole UtA and cultured UtA smooth muscle cells isolated from pregnant women [40]. Studies have shown that the pregnancy-dependent increase in β1 subunit expression was dependent on estrogen activity [41] and epigenetic regulation of the KCNMB1 gene promoter site, Sp1, via a decrease in CpG methylation [42]. This pregnancy-induced epigenetic regulation of β1 subunit expression was associated with an increased expression of the demethylase ten-eleven translocation methylcytosine dioxygenase 1 [43]. In addition, mice lacking the γ1 subunit showed a reduced rise in UtA diameter at late pregnancy stages and diminished vasoconstrictor responses evoked by the BK_Ca_ blocker IbTX [37]. Overall, these studies suggest that alterations in the BK_Ca_ auxiliary subunits contribute to the characteristic rise in BK_Ca_ activity, increase in UtA diameter, and decrease in UtA myogenic tone occurring during pregnancy.

Besides their interaction with BK_Ca_ auxiliary subunits, BK_Ca_ channels are also regulated by intracellular signaling [44]. Protein kinase C (PKC), protein kinase G (PKG), and other cell signaling pathways have been shown to affect the activation of BK_Ca_ channels in the UtA in a pregnancy-dependent manner. PKC reduces BK_Ca_ channel activity in vascular smooth muscle cells [45], and PKC activity is decreased during pregnancy in ovine and porcine UtA [46,47]. Furthermore, the inhibition of BK_Ca_ channels enhanced the PKC-dependent vasoconstriction in pregnant sheep UtA [48], suggesting that the pregnancy-reduced PKC activity increases BK_Ca_ in UtA smooth muscle cells and leads to an enhanced vasodilation of the UtA throughout pregnancy. Conversely, other studies have described that pregnancy increased the levels of cGMP in UtA and enhanced PKG and therefore BK_Ca_ activation [39]. An increase in Ca^2+^ sparks, released from the sarcoplasmic reticulum via ryanodine receptor types 1 and 2, directly regulated BK_Ca_ channel activation in UtA smooth muscle cells, leading to spontaneous transient outward currents [49,50]. These authors also observed that a lack of ryanodine receptors 1 and 2 decreased BK_Ca_ β1 subunit expression and the interaction between BK_Ca_ α and β1, thus indirectly regulating BK_Ca_ channel activity [50].

Extracellular factors have also been described to modulate BK_Ca_ channels during pregnancy [44]. The calcitonin gene-related peptide (CGRP), a neuropeptide widely distributed throughout the central and peripheral nervous systems, is a potent vasodilator [51]. The CGRP concentration increases throughout pregnancy, is positively correlated with birth weight, and is reduced in pre-eclamptic women [52,53]. UtA vasodilatory responses to CGRP via activation of the CGRP receptor A [54] were reduced by blocking BK_Ca_ channels with TEA [55,56]. Another vasoactive peptide, adrenomedullin (ADM), whose levels are increased during pregnancy [57,58], was shown to vasodilate pregnant rat UtA in vitro via BK_Ca_ channel activation [59]. In pregnant mice, supplementation with melatonin, a peptide released by the pineal gland, and, during pregnancy, by the placenta [60], restored ACh-dependent UtA vasodilation that was reduced by blocking BK_Ca_ channels with IbTX [61]. Recently, another known vasodilator, hydrogen sulfide (H_2_S), has been described to vasodilate human UtA via the activation of BK_Ca_ channels [40]. H_2_S production is enhanced by pregnancy in sheep and human UtA [62,63]. Using an H_2_S donor, Li et al. observed an increase in K^+^ currents in UtA smooth muscle cells and relaxation of UtA rings from pregnant women [40]. These effects of H_2_S were reduced by IbTX preincubation and were independent of Ca^2+^ signaling [40]. H_2_S also augmented BK_Ca_ currents when applied to outside-out patches, suggesting that H_2_S could directly activate BK_Ca_ channel [40], although the probable mechanism remains unknown.

### 2.2. BK_Ca_ Channel Impaired Function in Pregnancy Complications

Chronic hypoxia, one of the most potent factors acting to impair vascular function and pregnancy outcome, affects BK_Ca_ channel function. Exposure to chronic hypoxia augmented UtA vascular tone in pregnant animals and increased PKC activity [48,64], with a concomitant reduction in BK_Ca_ activity [48]. Reactive oxygen species have also been described to reduce the BK_Ca_-induced vasodilation of pregnant UtA from hypoxic-exposed sheep through a mechanism that involves the inhibition of steroid hormones function and downregulation of BK_Ca_ β1 subunits [65,66]. As described above, the epigenetic pregnancy-dependent demethylation of the β1 gene promoter leads to an increased expression of this auxiliary subunit [42]; it has been proposed that chronic hypoxia also affects this process by enhancing DNA methyltransferase activity, increasing the methylation of the β1 subunit promoter and decreasing its expression, resulting in reduced BK_Ca_-dependent currents and vasodilation in the UtA [67]. Moreover, the increased DNA methylation of the estrogen receptor α gene promoter by hypoxia downregulates its expression, leading to an increased UtA myogenic tone via reduced BK_Ca_ activity [68].

In addition, hypoxia increased the expression of microRNA 210 (miR-210), which represses ten-eleven translocation methylcytosine dioxygenase 1, leading to the hypermethylation of KCNMB1 and reduced BK_Ca_ channel activity in the UtA [69,70]. Notably, miR-210 has been described as a master hypoxamiR (hypoxia-induced microRNA) and its expression is directly increased by hypoxia-induced factors [71]. Furthermore, circulating and placental miR-210 levels were higher in pre-eclamptic women compared to healthy women [72,73,74], and miR-210 has been shown to regulate the expression of targets that contribute to impaired trophoblast function and reduced angiogenesis [75,76], both key features of pre-eclampsia. In placental tissue from pre-eclamptic women, the increased expression of miR-210 has also been associated with a reduction in the K^+^ channel modulatory factor 1, KCMF1 [77], which is related to cell proliferation, further suggesting miR-210 as a link between hypoxia and pregnancy complications. MicroRNAs can be released to the circulation in exosomes or bound to proteins, and it has been postulated that the increase in miR-210 in the plasma of pre-eclamptic women could be the result of an increased exosomal secretion [78]; thus, it is possible that miR-210 secreted through placental-derived exosomes to the maternal circulation reduces BK_Ca_-dependent vasodilation in the uterine vasculature. Further studies may elucidate the role of microRNA-containing exosomes in the regulation of ion channel function associated with pregnancy complications.

Altogether, the increase in BK_Ca_ activity appears to be highly regulated in the UtA during pregnancy and altered under hypoxic conditions. However, whether BK_Ca_ activity is similarly impaired under conditions of human pregnancy complications, such as pre-eclampsia and IUGR, remains to be determined.

Suggesting an important role for BK_Ca_ channel activity in such pregnancy complications are observations in stress-induced hypertensive mice during pregnancy. These hypertensive mice showed reduced levels of the BK_Ca_ β1 subunit in the UtA, and this reduction was partially reversed by supplementation with melatonin [61]. Functionally, the pharmacological blockage of BK_Ca_ channels with IbTX further reduced the already diminished ACh-dependent vasodilation in vitro in UtA from hypertensive pregnant mice compared to normotensive pregnant mice, and this effect of BK_Ca_ blockade was also prevented by melatonin supplementation [61]. These results highlight the role of impaired BK_Ca_ channel function in the pathophysiology of gestational hypertension.

### 2.3. SK and IK Channels in Healthy Pregnancies

SK and IK channels have been studied as important contributors to EDHF vascular responses and uteroplacental function during pregnancy. The pharmacological blockade of SK (apamin) and IK (charybdotoxin or TRAM-34) channels in human myometrial arteries reduced the EDHF-mediated vasodilation evoked by bradykinin [79]. A recent study showed that concurrently blocking SK and IK (with apamin and charybdotoxin, respectively) reduced the vasodilation induced by CGRP, ADM, and adrenomedullin 2 in pregnant human UtA [80]. Moreover, in UtA from both non-pregnant and late pregnant rats, blocking SK and IK channels abolished ACh-dependent vasodilation and prevented the decrease in intracellular Ca^2+^ levels in smooth muscle cells [81]. The SK-dependent component of ACh-induced UtA vasodilation was decreased in pregnant mice lacking relaxin, a hormone produced by the placenta with an important vasodilatory role in renal arteries, whereas the IK-channel-dependent component of ACh-evoked UtA vasodilation was augmented [82]. Furthermore, both SK and IK channels in the endothelium contributed to the ACh-dependent relaxation of UtA in non-pregnant buffaloes [83]. The IK channel blocker TRAM-34 prevented the dilation of non-pregnant and pregnant rat radial UtA (downstream of main UtA) induced by the activation of the transient receptor potential vanilloid type 3 ion channel [84]. In addition, activating IK channels with the channel opener SKA-31 elicited larger vasodilation in intact vessels from pregnant rats compared to non-pregnant rats or pregnant rats but with their endothelium removed [84]. Altogether, these findings suggest that SK and IK channels are important mechanisms for enhancing EDHF-mediated vasodilation in UtA. In ovine UtA, pregnancy also increased the SK-dependent vasodilation; however, this effect seems to be mediated by SK2 and SK3 channels predominantly expressed in the smooth muscle, since the removal of the endothelium did not change the vasodilation induced by an SK channel opener [85]. Using a different approach, another study showed that treating human uterine microvascular endothelial cells with serum from pregnant women compared to non-pregnant women increased the plasma membrane localization of SK3 and IK channels via a decreased caveolin-dependent internalization of SK3 and IK proteins [86].

### 2.4. SK and IK Channels in Pregnancy Complications

As described above, serum from pregnant women increases endothelial cell SK3 and IK channel plasma membrane localization compared to cells treated with serum from non-pregnant women. However, the localization of these channels in the plasma membrane was reduced in a clathrin- and caveolin-dependent manner when the endothelial cells were treated with serum from pre-eclamptic women [86]. The findings of Choi and colleagues indicate a role for internalization processes in the regulation of normal endothelial cell excitability and, in turn, vascular function, whereas an increased internalization of these channels may lead to the characteristic endothelial dysfunction observed in pre-eclampsia [87].

Similar to BK_Ca_ channels, chronic hypoxia also reduced the activity of SK channels. In a sheep model of pregnancy under hypoxic conditions, the expression of both SK2 and SK3 channel subtypes was reduced in pregnant UtA from animals exposed to chronic hypoxia [85]. Moreover, SK-dependent currents, vasodilatory responses, and a reduced myogenic tone were also abolished in UtA from pregnant sheep in hypoxic conditions compared to pregnant sheep in normoxic conditions [85], suggesting an impaired function of SK channels in the uterine vascular responses under hypoxic conditions during pregnancy.

Using a rat model of diabetes mellitus during pregnancy, Gokina et al. found that endothelial IK-dependent, but not SK-dependent, vasodilatory responses and ionic currents were reduced in radial arteries from diabetic pregnant rats compared to controls [88]. These observations suggest that IK channels are important contributors to the impaired uterine endothelial function, which has been observed in human pregnancies complicated by diabetes [89].

Increased testosterone levels during pregnancy can also be detrimental to fetal development. High levels of testosterone and a reduced UtA blood flow were observed in human pregnancies affected by polycystic ovary syndrome [90]. Rats treated with testosterone during pregnancy showed a decreased pup weight, increased UtA responses to vasoconstrictors, and reduced UtA vasodilatory responses compared to control animals. The lower vasodilatory response was partially attributable to a reduced EDHF component and decreased endothelial SK3 channel expression [91], suggesting a role for these channels in the UtA dysfunction induced by elevated testosterone levels.

Notably, mice overexpressing SK3 channels showed a reduction in the G-protein-mediated UtA contractile response and an increased fetal loss compared to wild-type animals [92]. Thus, special attention should also be given to the observations that exaggerated SK channel expression and activity could result in adverse pregnancy outcomes.

## 3. ATP-Sensitive K^+^ Channels

ATP-sensitive K^+^ (K_ATP_) channels are expressed in many tissues, especially vascular smooth muscle, and are composed of the inwardly rectifying K^+^ channel family (Kir6.1 and Kir6.2), which forms the ion-conductive pore, and the regulatory ATP-binding sulfonylurea receptors (SUR1, SUR2A, and SUR2B). At least two of each of these subunits form a functional channel. In myometrial and vascular smooth muscle, Kir6.1/SUR2B and Kir6.2/SUR2B are the combinations that result in functional K_ATP_ channels [93,94] whose activity is enhanced by intracellular ADP acting on SUR2B and inhibited by intracellular ATP acting on Kir6.1 and Kir6.2 [95,96]. K_ATP_ channel activation in vascular beds increases vasodilation and blood flow [22], positioning these channels as important integrators of cellular metabolism with blood flow. This concept has been studied using the metabolic inhibitor, Na-azide, which decreases cellular ATP concentrations and increases K_ATP_ channel basal currents [97]. Adenosine, prostacyclin, and β-adrenergic agonists are among several vasodilators that act to enhance K_ATP_ channel activity through cyclic-AMP (cAMP) and protein kinase A (PKA) signaling pathways, ultimately resulting in membrane hyperpolarization and vasorelaxation [24,98]. Angiotensin II (Ang II), serotonin, endothelin-1, norepinephrine, and α-adrenergic agonists are vasoconstrictors that decrease K_ATP_ channel activity through the activation of PKC, resulting in membrane depolarization and contraction [24,99,100,101,102]. K_ATP_ channel activity and function have been studied using several different channel openers and blockers, such as the K_ATP_ channel openers pinacidil and diazoxide, as well as glibenclamide, which is a potent K_ATP_ channel blocker [103].

### 3.1. K_ATP_ Channels in Healthy Pregnancies

The K_ATP_ channel has been shown to play a major role in the modulation of membrane potential by the hyperpolarization and reduction in L-type Ca^2+^ channel activity leading to a decrease in intracellular Ca^2+^ concentrations, causing an overall reduction in vascular tone. In sheep, the K_ATP_ channel opener, diazoxide, induced UtA vasorelaxation by decreasing intracellular Ca^2+^ concentrations and attenuating phenylephrine-induced vascular contractions [104]. These effects were observed in both pregnant and nonpregnant sheep but were more potent in pregnant UtA than in nonpregnant UtA [104]. Similarly, the K_ATP_ channel opener pinacidil has been shown to oppose norepinephrine-induced vasoconstrictions in non-pregnant human UtA [105]. Moreover, blocking K_ATP_ channels with glibenclamide in vivo evoked a larger increase in blood pressure and systemic and uterine vascular resistance in pregnant animals compared to non-pregnant animals [106]. Ang II infusion also induced an increase in systemic and uterine vascular resistance that was further augmented by treatment with glibenclamide in non-pregnant and largely in pregnant guinea pigs [106], suggesting that K_ATP_ channels are important regulators of systemic and uterine vascular responses during pregnancy. In a state of metabolic demand, such as is seen during pregnancy, ATP levels decrease because of rapid consumption, which facilitates the opening of the K_ATP_ channel due to a lack of inhibition at the Kir subunit family [96]. Hence, the decreased ATP levels may be another contributor to the enhanced K_ATP_ channel activity during pregnancy.

K_ATP_ channels can also be regulated by other factors. For instance, CGRP activates K_ATP_ channels in endothelial and smooth muscle cells [107]. CGRP increases during pregnancy, the magnitude of which is directly correlated to fetal weight [53]. In the uterine vasculature of pregnant rats, CGRP activated K_ATP_ channels to induce vasodilation [55,56]. CGRP has also been shown to relax norepinephrine-constricted human UtA, and this vasodilation was inhibited by glibenclamide [105]. Taken together, these results indicate that the K_ATP_ channel is a key effector in the CGRP-mediated relaxation of UtA and that this relaxation is increased by pregnancy.

### 3.2. K_ATP_ Channel Impaired Function in Pregnancy Complications

K_ATP_ channel dysfunction contributes to the pathological conditions of hypertension, diabetes, obesity, hypercholesterolemia, atherosclerosis, and ischemia [22,108]. The K_ATP_ channel has been shown to play a role in the hypoxia-mediated reduction in UtA vasodilation. In pregnant sheep under normoxic conditions, the pharmacological opening of K_ATP_ channels decreases intracellular Ca^2+^ levels and increases UtA vasorelaxation, whereas pregnant animals maintained under long-term hypoxic conditions showed decreased K_ATP_-dependent vasodilation [104], highlighting a role of these channels in the hypoxia-reduced UtA vasodilation. In vascular smooth muscle, K_ATP_ channels were inhibited by several vasoconstricting hormones and neurotransmitters through the activation of PKC. In the UtA, PKC activity is reduced in normal pregnancy but enhanced in the state of long-term hypoxia [64]; thus, the pregnancy-mediated increase and chronic hypoxia-mediated decrease in K_ATP_ channel activity may be regulated via alterations in PKC signaling.

K_ATP_ channels have also been described in fetal vessels. Yin and collaborators found that the mRNA and protein expression of the K_ATP_ channel subunit SUR2B were lower in the umbilical artery from cases of severe pre-eclampsia than in healthy pregnancies [109]. These observations imply an association between reduced K_ATP_ activity, through a diminished SUR2B expression, and the development of severe pre-eclampsia [109]. SUR2B mRNA and protein expression levels were also lower in early-onset severe pre-eclampsia than in late-onset severe pre-eclampsia, suggesting that the degree of the reduction in SUR2B expression may be related to the occurrence of severe pre-eclampsia [109], perhaps via K_ATP_ channel inhibition and reduced vasodilation.

Pinacidil-evoked K_ATP_ channel currents and vasodilation are reduced in the umbilical artery smooth muscle of pregnant women diagnosed with gestational diabetes mellitus (GDM) compared to a healthy pregnancy cohort [110]. mRNA and protein levels of K_ATP_ subunits Kir6.1, Kir6.2, and SUR2B were also decreased in GDM compared to the healthy control group [110]. However, the responses to adenosine or PKA activation, which activates K_ATP_ channels, did not differ, indicating that impaired K_ATP_ channel activity and vasorelaxation were due to downregulated K_ATP_ channel expression rather than changes in associated signaling [110]. A reduction in Kir6.1 subunit expression has also been observed in umbilical veins of women affected by pregnancy-induced hypertension and GDM [111]. In functional studies, mice with Kir6.1 deletions exhibited hypertension [112], whereas the gain-of-function in this subunit has been linked to hypotension [113], further suggesting its role in the regulation of vascular tone and thus blood pressure control. Furthermore, blocking K_ATP_ channels with glibenclamide increased systemic blood pressure in normotensive pregnant rats but did not further increase it in spontaneously hypertensive rats [114]. Thus, the study by Lima and colleagues suggests an impaired regulation of blood pressure by the K_ATP_ channel in pregnancies affected by hypertension [114].

IUGR and pre-eclampsia have been associated with decreased CGRP levels in maternal and fetal circulation and reduced placental CGRP expression [53]. Because CGRP is a known vasodilator and modulator of K_ATP_ activity in UtA from healthy pregnancies [55,56], a decrease in CGRP could impair the normal uteroplacental blood flow and could be associated with the etiology of IUGR and pre-eclampsia. However, future studies are needed to determine whether reduced CGRP levels lower K_ATP_ channel responses in the uterine vasculature of pregnancy complications.

## 4. Voltage-Gated K^+^ Channels

Voltage-gated K^+^ (Kv) channels are important regulators of membrane potential. They allow K^+^ efflux in response to the depolarization of the membrane. The mammalian Kv channel family is vast, with 12 distinct types (Kv1–12) forming homomers or heteromers [115]. The α subunits form functional channels, which can also be associated with auxiliary proteins [116].

In smooth muscle cells, Kv channels are activated by depolarization and provide a negative feedback mechanism that limits contraction [24,117]. Therefore, the constitutive activation of these channels plays an important role in maintaining myogenic tone in blood vessels. Vasoconstriction results from the inhibition of Kv channels in vascular smooth muscle cells via vasoconstrictors such as phenylephrine and Ang II [118,119,120]. In contrast, known vasodilators such as NO and H_2_S contribute to the activation of Kv channels in vascular smooth muscle [121,122,123,124]. These channels can also be activated under the conditions of acidosis or hypoxia, or by the influence of anticontractile substances derived from peripheral vascular adipose tissue [125,126,127].

### 4.1. Kv Channels in Healthy Pregnancies

The physiologic role of Kv channels in uterine vasculature has not been well characterized. Initial studies in non-pregnant sheep found that the in vivo infusion of 4-aminopyridine (4-AP), a selective Kv blocker, did not alter the basal uterine blood flow but did reduce the estradiol-induced rise in uterine blood flow [8]. K^+^ currents in cultured human UtA smooth muscle cells showed a predominant component attributable to Kv1 channels, whereas protein expression profiles showed the presence of Kv1.2, Kv1.3, Kv1.5, Kv3.4, Kv4.2, and Kv4.3 subtypes in human UtA tissue [128].

The effect of blocking Kv channels in the UtA has been studied in vitro. For instance, ACh-dependent vasodilation is only partially reduced by 4-AP in UtA from pregnant mice [61]. Conversely, exposure to 4-AP enhanced a pressure-evoked increase in intracellular Ca^2+^ concentrations and myogenic tone in radial UtA, and this effect was greater in non-pregnant rats than late-pregnant rats [129]. This, coupled with the significant decrease in currents through Kv channels in radial UtA smooth muscle cells from late-pregnant rats versus non-pregnant rats, indicates that a reduction in Kv channel activity in smooth muscle cells of small, resistance-size uteroplacental arteries contributes to vasoconstriction in late pregnancy [129]. Additionally, in non-pregnant guinea pigs, ACh-dependent UtA relaxation was not affected by incubation with 4-AP, nor by other K^+^ channel blockers such as TEA, glibenclamide, or apamin [130]. Consequently, a role for Kv channels in ACh-evoked vasodilation in UtA from pregnant mice, a greater effect of a Kv blocker in enhancing myogenic tone in radial arteries from non-pregnant rats than pregnant rats, and a lack of effect in blocking Kv channels in the ACh-dependent UtA vasodilation in non-pregnant guinea pigs highlight the potential diverse roles of Kv that may differ by species, vessel size, and/or pregnancy state.

The Kv channel subtype Kv3.4, expressed in the human UtA, has been associated with the proliferation of UtA vascular smooth muscle cells in culture [128]. Thus, the direct inhibition of this channel, as well as an increase in extracellular K^+^ to mimic Kv channel inhibition, resulted in a failure in the vascular smooth muscle cells to enter mitotic growth phases required for proliferation [131], indicating that smooth muscle cell hyperpolarization mediated by Kv channels is directly correlated with vascular smooth muscle proliferation.

### 4.2. Kv Channels in Pregnancy Complications

The role of Kv channels in the maternal vasculature of pregnancy complications has not been well described, but there are some studies on its role in the fetoplacental circulation. Hypoxia selectively reduced Kv currents measured by the patch clamp technique in smooth muscle cells from peripheral fetoplacental arteries and induced vasoconstriction in placental cotyledons [132]. Another study has shown an increase in the mRNA expression of Kv2.1 in chorionic plate veins from fetal growth-restricted pregnancies compared to healthy controls, whereas Kv9.3 levels were not altered [133].

## 5. Therapeutic Perspectives

Several characteristics of K^+^ channels make them relevant therapeutic targets for the prevention or treatment of pregnancy complications: (1) their important contribution to the vascular adaptation to pregnancy and their impaired function in pregnancy complications, (2) their role as end-point effectors for many vasodilators or vasoconstrictors, which is beneficial from a clinical point of view, as the modulation of their activity could lead to strong physiological responses, (3) the selective activation of these channels under different conditions (i.e., pregnancy, hypoxia, ischemia, etc.), and (4) their differential expression in distinct cell/tissue types. Currently, several therapeutic interventions using modulators of K^+^ channels during pregnancy are being assessed in clinical trials [134]. For instance, K_ATP_ channel blockers, such as glibenclamide, are being tested to alleviate the effects of GDM on fetal growth and adverse pregnancy outcomes. Furthermore, the K_ATP_ channel agonist nicorandil is currently being assessed for the prevention of preterm labor. As reviewed here and summarized in Table 1, multiple K^+^ channel openers and blockers have specific effects in the uterine vasculature. The role of K^+^ channels in vascular adaptation to pregnancy and their importance as modulators of a wide variety of signals may position K^+^ channels as good candidates for therapeutic intervention.

## 6. Conclusions

Pregnancy induces major changes in maternal uterine vasculature that enable a rise in UtA blood flow to the uteroplacental circulation required to meet the demand for nutrients and oxygen for normal fetal development. K^+^ channels have been widely described as effectors for multiple vasodilators, many of which are positively regulated during pregnancy. Conversely, in cases of pregnancy complications or under harmful conditions (such as hypoxic conditions), K^+^ channels in uterine vessels are downregulated and their activity is impaired. As reviewed here, the specific localization of different K^+^ channels, their selective gestational upregulation, and their modulatory role in the vascular adaptation to pregnancy make them potential therapeutic targets. However, since complications of pregnancy are likely the result of multiple factors (maternal, fetal, placental, genetic, environmental, etc.), additional research is needed for addressing the complex factors involved to prevent or treat pregnancy complications associated with dysfunctional uterine vascular adaptation to pregnancy. We expect that this comprehensive literature review will help researchers to identify the complex nature of the vascular adaptations to pregnancy and use this review as a tool for the development of new approaches.

## Figures and Tables

**Table 1:** K^+^ channel openers and blockers, and their effect on uterine vasculature.

Drug	Mechanism	Effect on Uterine Vasculature	Ref.
NS-1619	BK_Ca_ opener	Relaxed pregnant UtA	[85]
Tetraethylammonium	BK_Ca_ blocker	Decreased UtA blood flow	[38]
		Increased UtA myogenic tone, reduced BK_Ca_-evoked currents	[36]
		Diminished ADM-induced UtA vasodilation	[59]
		Reduced CGRP-dependent UtA vasodilation and prevented CGRP-induced decrease in UtA perfusion pressure	[55,56]
Iberiotoxin	BK_Ca_ blocker	Decreased late pregnant UtA diameter in wild-type but not BK_Ca_ γ1 subunit knockout mouse	[37]
		Reduced ACh-elicited UtA vasodilation	[61]
		Diminished H_2_S-induced UtA vasodilation	[40]
NS-309	SK opener	Induced vasodilation in non-pregnant UtA	[83]
Apamin	SK blocker	Reduced EDHF-dependent myometrial artery vasodilation (in combination with TRAM-34 or charybdotoxin)	[79]
		Decreased UtA vasodilation induced by CGRP, ADM, and ADM2 (in combination with charybdotoxin)	[80]
		Diminished ACh-induced UtA vasodilation and increased Ca^2+^ levels in smooth muscle cells (in combination with charybdotoxin)	[81]
		Reduced ACh-induced vasodilation in non-pregnant UtA (with or without TRAM-34)	[83]
		Relaxed pregnant UtA	[85]
SKA-31	IK opener	Induced vasodilation in radial UtA (greater in pregnant than non-pregnant)	[84]
TRAM-34	IK blocker	Prevented ACh-induced vasodilation in non-pregnant UtA (with or without apamin)	[83]
		Decreased TRPV3-evoked vasodilation in radial UtA	[84]
Charybdotoxin	SK/IK blocker	Attenuated EDHF-dependent myometrial artery vasodilation	[79]
Diazoxide	K_ATP_ opener	Induced vasodilation of UtA, decreased Ca^2+^ levels	[104]
Pinacidil	K_ATP_ opener	Reduced PE-dependent UtA vasoconstriction	[105]
Glibenclamide	K_ATP_ blocker	Increased uterine vascular resistance, exacerbated the increase in uterine vascular resistance induced by AngII	[106]
4-Aminopyridine	Kv blocker	Partially reduced ACh-induced vasodilation of UtA	[61]
		Increased myogenic tone and Ca^2+^ levels in radial UtA	[129]
BDS-I	Kv3.4 blocker	Prevented proliferation in UtA smooth muscle cells	[131]

NS-1619, 1,3-dihydro-1-[2-hydroxy-5-(trifluoromethyl)phenyl]-5-(trifluoromethyl)-2H-benzimidazol-2-one; NS-309, 6,7-dichloro-1H-indole-2,3-dione 3-oxime; SKA-31, naphtho[1,2-d]thiazol-2-ylamine; TRAM-34, 1-[(2-chlorophenyl)diphenylmethyl]-1H-pyrazole; BDS-I, blood-depressing substance-1; UtA, uterine artery; ADM, adrenomedullin; CGRP, calcitonin gene-related peptide; ACh, acetylcholine; H_2_S, hydrogen sulfide; EDHF, endothelial-derived hyperpolarizing factor; ADM2, adrenomedullin 2; TRPV3, transient receptor potential vanilloid 3; PE, phenylephrine; AngII, angiotensin II.
